# Postmortem brain donations vs premortem surgical resections for glioblastoma research: viewing the matter as a whole

**DOI:** 10.1093/noajnl/vdab168

**Published:** 2021-11-18

**Authors:** Cassandra P Griffin, Christine L Paul, Kimberley L Alexander, Marjorie M Walker, Hubert Hondermarck, James Lynam

**Affiliations:** 1 School of Medicine and Public Health, University of Newcastle, Callaghan, New South Wales, Australia; 2 Hunter Cancer Biobank: NSW Regional Biospecimen and Research Services, University of Newcastle, Callaghan, New South Wales, Australia; 3 Hunter Cancer Research Alliance, University of Newcastle, Newcastle, New South Wales, Australia; 4 Hunter Medical Research Institute, Newcastle, New South Wales, Australia; 5 Priority Research Centre Cancer Research, Innovation and Translation, University of Newcastle, New South Wales, Australia; 6 Priority Research Centre Health Behaviour, University of Newcastle, New South Wales, Australia; 7 Neurosurgery Department, Chris O’Brien Lifehouse, Camperdown, New South Wales, Australia; 8 Brainstorm Brain Cancer Research, Brain and Mind Centre, The University of Sydney, New South Wales, Australia; 9 Neuropathology Department, Royal Prince Alfred Hospital, Camperdown, New South Wales, Australia; 10 School of Biomedical Sciences and Pharmacy, University of Newcastle, Newcastle, New South Wales, Australia; 11 Department of Medical Oncology, Calvary Mater, Newcastle, New South Wales, Australia

**Keywords:** biobanking, brain, cancer, glioblastoma, postmortem

## Abstract

There have been limited improvements in diagnosis, treatment, and outcomes of primary brain cancers, including glioblastoma, over the past 10 years. This is largely attributable to persistent deficits in understanding brain tumor biology and pathogenesis due to a lack of high-quality biological research specimens. Traditional, premortem, surgical biopsy samples do not allow full characterization of the spatial and temporal heterogeneity of glioblastoma, nor capture end-stage disease to allow full evaluation of the evolutionary and mutational processes that lead to treatment resistance and recurrence. Furthermore, the necessity of ensuring sufficient viable tissue is available for histopathological diagnosis, while minimizing surgically induced functional deficit, leaves minimal tissue for research purposes and results in formalin fixation of most surgical specimens. Postmortem brain donation programs are rapidly gaining support due to their unique ability to address the limitations associated with surgical tissue sampling. Collecting, processing, and preserving tissue samples intended solely for research provides both a spatial and temporal view of tumor heterogeneity as well as the opportunity to fully characterize end-stage disease from histological and molecular standpoints. This review explores the limitations of traditional sample collection and the opportunities afforded by postmortem brain donations for future neurobiological cancer research.

More than two-thirds of adults diagnosed with glioblastoma (GBM) will die within 2 years of diagnosis.^1^ In the pediatric setting, brain malignancies are both the most lethal and most common of all solid tumors.^2^ While recent data has suggested an improvement in short-term survival, long-term survival remains poor and this is largely attributed to an incomplete understanding of brain tumor biology and limited access to high-quality biological samples for research.^3^ Whilst the importance of postmortem brain samples for research into neurodegenerative disease is well appreciated,^4^ by comparison, this is not true for primary brain cancer.

Postmortem brain donation is indispensable for neuropathological research and is a major contributor to the understanding of the molecular and cellular pathways underpinning neurological disease.^5,6^ Characterization at autopsy and analysis of pathological hallmarks provides a unique and macroscopic overview of disease presentation and progression that cannot be achieved through the analysis of smaller tissue samples obtained during surgical procedures,^7^ nor through radiological studies captured premortem.^8^ This becomes even more pertinent when one considers the spatial heterogeneity of brain tumors. Postmortem tissue samples provide essential insight into brain tumor pathophysiology, enabling identification of targets for drug development and biomarkers for early detection and prevention of disease.^9^ Perhaps the greatest contribution afforded by postmortem samples is their representation of end-stage disease and further mutational changes post resection of recurrent disease, illustrating ways in which tumors adapt to selective pressures imposed by therapeutic approaches—ultimately resulting in a convergent evolution towards treatment resistance. Demonstration of this can be seen in the work of Kim et al (2015)^10^ who assessed primary and post treatment recurrent samples using next-generation sequencing (NGS) to reconstruct the genomic profile of therapy-resistant tumor cells. Their work identified divergent recurrences that shared few genetic alterations with the primary tumor. With the emergence of third and fourth-line treatments such as lomustine and bevacizumab, assessment beyond initial recurrence, and following subsequent treatment, is essential for further characterization. Unfortunately, such efforts are hampered by limited opportunities for surgical resection and often a rapid clinical decline. In providing a sample of the final and fatal iteration of disease, postmortem tissue affords detailed investigations into the mechanisms and molecular pathways of treatment resistance and tumor evolution. These insights are beyond that which can be viewed in recurrent samples often obtained months prior to death and prior to the withdrawal of treatment.

This review highlights the importance and unique contribution afforded to research by the provision of postmortem brain tumor samples.

## Postmortem Brain Tissue in Neurodegenerative and Neuropsychiatric Disease

It is well recognized that in neurological, neurodevelopmental, and neuropsychiatric disorders there is no substitute for studying human brain tissue.^4^ Most brain diseases are complex entities and while animal models or cell culture methods can mimic some aspects of disease, human postmortem tissue remains essential in the advancement of our understanding.^11^ Given routine surgical resection or diagnostic sampling is not indicated or indeed practical in these diseases, access to human brain tissue samples is obtained exclusively in the postmortem setting. Research autopsies were first employed in the neurological and psychiatric disease space at the end of the 19th century. In the cancer setting, research autopsies were primarily responsible for Stephen Paget’s work proposing that metastatic disease demonstrates tissue trophism and specificity,^12^ reinforcing the importance of postmortem tissue for biomedical research.

The importance of postmortem specimens and their role in understanding neurodegenerative disease has been well documented, for example in Parkinson’s disease,^13^ Alzheimer’s,^14,15^ dementia^16,17^ and schizophrenia.^18,19^ A series of clinicopathological studies in the 1970s and 1990s examining postmortem brains was key to understanding the etiology and pathogenesis of Alzheimer’s disease, noting the cholinergic and vascular basis of cognitive deficits in Alzheimer’s^20^ and vascular dementia.^21^ As a result, most brain banking protocols have been developed for tissue use in these settings rather than to accommodate the specific variables associated with death due to primary brain cancer. Given the impetus to better interrelate neurobiological discovery with clinical practice, the role of postmortem tissue in brain cancer research is important and consideration of unique protocols and support for such programs is needed.

## What are the Limitations of Brain Cancer Research Samples Obtained Through Surgical Resection?

Weighing the risk of postoperative deficits with clinical benefit, particularly in high-grade brain tumor cases, remains challenging for neurosurgeons.^22^ While there is a growing trend towards supramaximal resection, lobectomy^23^ and resection of fluid-attenuated inversion-recovery (FLAIR) positive cortex surrounding contrast enhancement,^24^ the primary intention of surgical resection is to obtain diagnostic material, alleviate existing pathological deficit and avoid the introduction of new functional deficits. While increasing the scope of tumor resection may extend survival, there is cautioning against radical resection given the risk of new-onset postoperative neurological sequelae dramatically affecting function and quality of life.^22^ Histological heterogeneity and the inability to completely resect tumors due to the diffusely infiltrative nature of neoplastic cells is further cause for a conservative approach.^25^ This undermines any intention of obtaining designated research specimens, while the need to maximize the diagnostic potential of resected specimens often restricts the amount of sample available for research.

Tissue artifacts that may be caused by surgical equipment must also be considered when discussing the research utility of surgically resected brain tumor tissues, though it is worth noting that with the increasing demand for research specimens and an enduring emphasis on sample integrity for diagnosis, great care is taken to minimize surgical artifacts where possible. Potential artifacts include those from the Cavitron Ultrasonic Surgical Aspirator (CUSA) instrument that has been documented to increase edema resulting in minor alterations to tissue morphology,^26^ as well as side-cutting biopsy needles that can cause a band-like tissue compression and cause tumor tissues to appear hypercellular and spindle-like or mimic pseudopalisading cellular arrangements. This “peripheral compressing artifact” is particularly problematic as it can be confused with high-grade tumor features.^27^

Furthermore, obtaining spatially stratified tissue samples in the setting of aggressive, high-grade brain cancer is compromised by hemorrhage, brain shift, and subjective identification of “normal” brain for comparative study.^28^ Given recent work undertaken on surrounding tissues and necrotic areas associated with GBM growth^29^ and treatment, the sampling of spatially diverse and stratified tissue samples is not readily facilitated by surgery but is equally as imperative for research purposes. In the work of Iwadate et al.,^29^ immunohistochemical analysis indicated high expression of TGF-B and key epithelial-mesenchymal transition factor TWIST within pseudopalisading necrotic areas sampled from GBM tumors. Further analysis of these factors in postmortem tissues would facilitate additional regional sampling and extensive characterization of necrotic regions resulting from both tumor growth and treatment. Given Iwadate et al.’s (2016)^29^ data and its implications for anti-VEGF therapy to modulate hypoxic microenvironments and to potentiate radiotherapy effect, further characterization of pseudopalisading necrotic areas in postmortem tissues presents exciting prospects.

By nature, incomplete resection is discordant with the desire to capture a complete and comprehensive sample for research purposes and despite advancements in supramaximal resection, complete and global analysis of disease is not feasible outside the postmortem setting. Collecting tissue in a postmortem setting affords researchers access to complete samples that provide a comprehensive and complete picture of the tumor in situ.

For a subset of patients with inoperable tumors within the brainstem or other critical deep cortical structures, surgical sampling for research is not possible. This is the case for midline gliomas characterized by the H3 K27M mutation,^30^ which is especially prevalent in the pediatric population and in diffuse intrinsic pontine glioma (DIPG).^31^ Here, tiny biopsy samples are attainable only after recent developments in stereotactic biopsying.^32^ Postmortem specimens, however, have enabled pivotal research into understanding oncogenic signaling pathways and identifying candidate mediators of metastatic spread,^33,34^ including Hedgehog signaling in DIPG.^34^ The work of Lin et al (2018) on the characterization of a glioma subtype-specific inflammatory environment, through assessment of the secretome and concordant bulk and single-cell RNA sequencing, indicated that the inflammatory profile of the DIPG tumor microenvironment is fundamentally different to that of GBM carrying implications for immunotherapy-based treatments.^35^ Moreover, recent data relating to DIPG xenografts suggest that primary cultures established from autopsy samples are more likely to engraft into animals than those established from biopsy samples, with success rates of 47.4% and 86.7% respectively.^36^ These figures present a strong argument for the value of postmortem brain samples, particularly when one considers the potential for postmortem patient tumor-derived xenograft models to expedite the translation of new therapeutic agents.^34^

## Spatial and Topographic Heterogeneity—Can Biopsies Really Provide a Global and Comprehensive Picture?

One of the hallmarks of GBM is tumor heterogeneity, both within and between individual tumors, and this represents a major obstacle for effective treatment.^37^ While inter-tumor heterogeneity is best addressed by increasing the volume of available samples for research, discussions of intra-tumor heterogeneity are directly relevant within the scope of postmortem brain donation. Understanding brain tumor heterogeneity is crucial in elucidating the global mechanisms of, and overcoming, tumor recurrence.

Due to the clonal origin of most cancers, the process of tumorigenesis involves cancer stem cells generating progenies that are phenotypically diverse and include both mature stem cells that are capable of indefinite self-renewal and differentiating cells with limited proliferative potential. As was recognized by Soeda et al., there is no convincing evidence to suggest that only a single cancer stem cell is representative of patients tumors.^38^ Data obtained through fluorescence-activated cell sorting and cDNA-microarray analysis suggests that sub-clones within the same tumor exhibit variations in treatment sensitivity, metabolic characteristics, proliferative potential, and self-renewal.^38^ The marked heterogeneity of GBM tumors may be induced through dynamic differentiation and dedifferentiation processes, resulting in a highly plastic, diverse tumor phenotype.^39,40^

Molecular characterization plays a central role in broadening understandings of brain tumor biology and heterogeneity. As larger numbers of individual tumors are characterized at increasing molecular resolutions it is well recognized that there is substantial mutational heterogeneity within the same histopathological subtype.^41^ Heterogeneity as it applies to histological structures is well characterized,^42^ yet the complex interplay of cellular dynamics is less so—particularly as it relates to tumor recurrence and continued tumor evolution in response to treatment and/or changes in tumor microenvironments.^43^ Recent evidence demonstrates that GBM cells can hijack normal neuronal activity via paracrine signaling and direct electrochemical synaptic communication to support and promote tumor expansion.^44,45^ Understanding that neurons play a critical role in cancer progression,^46^ including the global neuronal context provided by assessment of the brain as a whole in the postmortem setting is crucial. Furthermore, as cancer neuroscience emerges as a distinct field,^47^ neuron-cancer cell crosstalk is increasingly appearing as a promising target for the treatment of brain cancer. Tumors are characterized at increasingly higher molecular resolution, and therefore heterogeneity is becoming more apparent even within histopathological subtypes^41^ and new classification systems will likely facilitate improvements in diagnosis and treatment.

With an appreciation for the vast intra-tumoral heterogeneity of GBM, it stands to reason biopsies grossly under sample and are insufficient for complete characterization. As cells infiltrate well beyond the radiological and indeed surgical limits of the tumor, GBM must be considered a systemic brain disease rather than a delineated tumor.^48^ The work of Burger et al.^48^ cautions against the limitations of needle biopsy for grading of astrocytic neoplasms following a topographic assessment of postmortem GBM samples. Based on cellular densities and topographic distribution, three categories of neoplasms were defined, suggesting that progression from a differentiated neoplasm to a primarily undifferentiated neoplasm is common. Further assessment suggested that while de novo appearance of overt malignancy remains possible, in IDH mutant tumors, patterns of widespread necrosis also suggest the presence of a preexisting, better-differentiated neoplasm overcome by the anaplastic component. Postmortem tissue analysis is essential for the complete characterization and assessment of GBM tumor heterogeneity and treatment-resistant progression, which holds particular relevance to the peritumoral brain zone (PBZ) where up to 90% of tumor recurrences occur.^49^ Given the PBZ comprises macroscopically normal brain tissue peripheral to the tumor mass, it frequently represents the margin of surgical resection. Despite being aligned with a microenvironment that possesses specific properties contributing to tumor heterogeneity, PBZ studies are limited, perhaps due to a scarcity of samples and the desire to avoid resecting macroscopically normal brain—an obstacle overcome by the facilitation of postmortem tissue donation.

## Temporal Heterogeneity—Single Snapshots in Time, What do Biopsies Miss?

Aligned with an understanding of intra-tumor heterogeneity is the notion that molecular alterations may be present in only a subset of tumor cells. This has additional significance when one considers the implications of treatment effect, treatment resistance, and/or disease progression leading to selective expansion or regression of tumor cell subpopulations. In the setting of recurrent progression there are further complexities in the space of temporal heterogeneity and delineating the pathophysiology of matched pairs of serial brain tumor specimens (primary and recurrent tumors) may shed light on the temporal sequence of molecular and cellular changes resisting or responding to therapy.

Given the rarity of appropriate surgical specimens specifically collected for research purposes, coupled with reduced frequencies of repeat neurosurgeries, recurrent samples are difficult to access for research.^50^ Data vary on the rates of secondary surgery for recurrent GBM, with some reports documenting that the percentage of patients undergoing secondary surgeries may be as low as 10–30% due to decreased performance status measures (such as the Karnofsky score) or anatomical location.^51,52^ Complicating this further is the presence of radiation necrosis and other treatment-related brain changes in specimens recovered from GBM recurrence surgeries that may have been mistaken for actively proliferating tumor during MRI surveillance. While multiple specimens representing important clinical timepoints such as primary disease, recurrence, and end stages are needed for critical translational research, tumor specimens are often not captured beyond the initial primary resection. Comparison of these timepoints allows for a complete assessment of temporal tumor heterogeneity post all medical and palliative intervention. Importantly, this would allow investigations of how tumor tissues change in response to second and third-line therapies. This adds further weight to the value of establishing systems for making postmortem specimens available for research.

The emergence of a hyper-mutational phenotype in treatment-resistant GBM recurrent tumors is well documented.^53^ Variations in MGMT methylation status, EGFR variants, TP53, MLH1, MSH6, PSM2, IDH1R132, TIMP3, CDKN2A, RB1, and NDRG2 provide specific examples of characterized mutational changes between primary and recurrent tumors.^54,55^ One such example of this is in the work of Nickel et al^41^ and their assessment of a patient with multiple incidences of recurrent GBM.^41^ Their data identified the rise of a subclonal population with a mutation in the tumor suppressor gene “PTEN” present in 50% of analyzed cells in the primary tumor sample. In a separate subclonal population, a PIK3CA mutation was identified and following first-line treatment both mutations were again identified in mutually exclusive populations. Following second line treatment, however the PIK3CA mutation was characterized as having acquired a hypermutator phenotype, accompanying three newly observed phenotypes.

Varying reports of mutational changes after second and third tumor recurrences highlights a potential oversight of the impact of temporal heterogeneity in studies using surgical specimens.^56^ Postmortem samples and their representation of end-stage disease following the withdrawal of treatment and supportive interventions allows for a complete characterization and investigation of all mutational and evolutionary events. This was demonstrated by Wakabayashi et al. in their investigation of cationic liposome-mediated interferon-beta gene transfer therapy for high-grade glioma, a treatment previously shown to induce experimental glioma regression.^57^ Here, altered gene expression patterns related to apoptosis, angiogenesis, and immune response pathways were identified in tumors sampled two weeks after the gene therapy trial. Histological examinations at autopsy revealed dramatic changes post treatment, including necrotic changes and decreased CD34 immunoreactive vessels.^57^ This application of autopsy samples validated surgical findings but also extended analysis beyond traditional study conclusion and depicted end-stage disease, facilitating specific assessment of the vector injected area and surrounding tissues for comparative purposes.

## Can we Truly Understand Brain Tumor Biology Without a Comprehensive Picture of the Tumor Microenvironment (TME)?

A thorough understanding of the functions and properties of the TME is essential to obtain a complete picture of the complexities of brain tumor biology. As outlined above, postmortem brain tissue studies have generated substantial knowledge of the contributions of the extracellular matrix, specific brain resident cellular populations such as microglia, neurons, and astrocytes, as well as the vasculature to the pathophysiology of neurodegenerative, neuropsychiatric, and other nonmalignant brain disorders. Understanding the immunological and vascular aspects of the TME may hold particular relevance for improving brain cancer outcomes, especially in the setting of immunotherapy.^3^ Such components include tumor-associated macrophages,^3^ regulatory T Cells,^58^ extracellular matrix components such as collagen,^59^ and heparin sulfate,^59^ secreted factors including chemokines and cytokines,^3^ neurotrophins,^58^ growth factors such as TGF-B^58^ and heparin-binding growth factors,^60^ small RNAs^61^ (sRNA) and extracellular vesicles (EVs).^62^ Given the challenges posed by temporal heterogeneity, assessment of the TME postmortem provides a unique opportunity to characterize immunological factors and identify targets for therapy at the most advanced stage of disease, while also understanding the molecular and histological changes caused through treatment-related effects and toxicities. Complementing this, assessment and profiling of TME components and the spatial heterogeneity between tumoral regions and the wider microenvironment further necessitates the need for a global representation of disease.

GBM progression is influenced by a unique self-sustaining, tumor-supportive microenvironment, in which EVs are increasingly shown to play a central role. EVs are membranous particles (e.g. “exosomes” and “microparticles”) that are utilized by cells for intracellular communication by selectively packaging molecules and delivering protected cellular information through the extracellular milieu to neighboring and distant cells.^62^ EVs are integral to glioma-cell signaling; GBM-EVs transfer oncogenic material to induce cell transformation,^63^ therapy resistance,^64^ influence endothelial cells to promote angiogenesis,^65^ mediate immune evasion^66^ and maintain intra-tumoral heterogeneity.^67^ Postmortem tissues provide a unique source of EVs “locked” in the TME and a growing body of evidence exists for this, particularly in the neurodegenerative disorders field. miRNA profiling of postmortem brain tissue EVs and corresponding analyses in EVs from patient blood samples has provided insight into Alzheimer’s disease pathophysiology as well as the development of a potential early diagnostic blood test.^68,69^ This is yet another example of the research potential afforded by fresh frozen, postmortem tissues as research foci expand beyond that which can be assessed in FFPE tissues.

Free or EV-cargoed sRNA, including microRNA (miRNA) species, target genes involved in glioma-genesis, tumor growth, proliferation, post transcriptional regulation of anti-oncogenes, and apoptosis.^70,71^ Given the potential application of miRNAs as therapeutics and biomarkers for companion diagnostics, a global representation of GBM disease is essential^72,73^ and reinforces the importance of collecting larger biospecimens that include specific tumor foci and surrounding “healthy” tissues.

A potential limitation, however, is the short half-life of miRNAs. Research protocols should accommodate possible rapid sRNA degradation with particular emphasis on ensuring samples are collected and stored with short postmortem interval for optimum sRNA preservation. This issue is discussed further below. By contrast, other TME components are documented to be relatively stable postmortem, including extracellular matrix components and growth factors, which are readily analyzed by a range of research applications including immunohistochemistry, molecular profiling, cell culture, and cell-based assays, and xenograft models. Such research strategies may reveal new insights into cellular and molecular mechanisms of GBM cell invasion to resolve novel targets that may hinder migratory and invasive capacity of these diffusely invasive tumors.^74,75^

In light of new approaches to understanding the TME and an increased focus on molecular profiles, it is essential that suitable samples are available for analysis. Postmortem brain donation, tissue processing, and preservation ensures optimal specimens are available for such investigations and that the full spectrum of disease and its relationship to the TME can be characterized—from diagnosis to death.

## What are the Considerations for Research Sample Fidelity When Considering Surgical and Postmortem Sample Collections?

While sample collections are often curated for “unspecified future research” planning and preparation to ensure that sample type is fit for investigation is a crucial factor. Both postmortem and surgical biopsy samples are largely stored as FFPE or fresh frozen tissue, though the latter is less common for surgical samples unless a designated research specimen is obtained. While FFPE is used for preserving tissue cytoarchitecture, the impact of formalin fixation on nucleic acids gains relevance as we move deeper in the omics era of research.^76^ Fragmentation, cross-linking, and the resultant Schiff bases (carbon-nitrogen double bonds) on free amino groups of nucleotides, leaves FFPE samples largely unsuitable for genomics, transcriptomics, and epigenomics modalities requiring high molecular weight nucleic acids for examination.^77^ With respect to NGS, numerous studies comparing FFPE and fresh frozen tissues have been documented, with a range of concordance rates reported.^78^ As discussed by Gao et al.,^78^ this may be due to variable factors such as primers, length of PCR product, tumor type, and testing methods and reinforces the need to consider research sample fidelity when planning tissue collections in both postmortem and surgical settings.

While DNA remains relatively stable between storage forms, total RNA, particularly mRNA, derived from FFPE samples exhibits greater levels of degradation than that obtained from fresh frozen specimens, potentially compromising biologically informative elements of inter-tumor heterogeneity.^79^ While there is a high degree of concordance between sample types at the variant and gene levels, important differences in tumor mutations have been identified, suggesting that sample fidelity should be a primary concern when curating collections.^78^ In their work assessing paired FFPE and fresh frozen tissue samples Gao et al. noted that the reads for fresh frozen tissues were significantly higher than FFPE tissues, 3,739 and 2,814 respectively, however, 226 variants were noted in FFPE tissues and only 221 in fresh frozen tissues. The authors hypothesize that the greater variant number identified in FFPE may be caused by DNA damage during the formalin fixation process.^78^ This is supported by Gallegos et al. who noted a 50% false-positive rate for PCR amplification in FFPE tissues compared to fresh frozen tissues in a lung cancer model.^80^

Table 1 provides an overview of emerging and enduring analytical techniques in brain cancer research, examining the impact of formalin fixation and concordance between results of FFPE and fresh frozen tissue analysis. Given the lack of comparative sample data exclusively within brain cancer models, work from other models has been referenced. This is a limitation of the body of evidence currently available and extrapolations must be considered critically.

**Table 1.** Important Techniques in Brain Cancer Research and Optimal Sample Types

Technology/Methodology	Compromised by formalin fixation?	Concordance % FFPE and FF data	Optimal sample	Study model
NGS—variant level^78^	Minimal	>94%	Fresh Frozen	Colorectal cancer
NGS—gene level^78^	Yes	>73%	Fresh Frozen	Colorectal cancer
miRNA LNA-based arrays^81^	No	~96%	Best Available	Mouse liver
miRNA oligo-array^81^	Yes	~56%	Fresh Frozen	
mRNA oligo-array^81^	Yes	<56%	Fresh Frozen	
DNA Methylation analysis (NGS)^82,83^	No	>99%	Best Available	Colorectal cancer
DNA Methylation analysis (NGS—threshold analysis)^82,83^	Yes	43-49%	Fresh Frozen	Colorectal cancer
RT-qPCR—gene expression^84^	Yes	~33%	Fresh Frozen	Breast cancer
Proteomics/phosphoproteomics—Mass spectroscopy^85–87^	Yes	70-90%	Fresh Frozen	Ovarian, breast cancer, canine tissue, glioblastoma
ctDNA—PCR^88^	NA	NA	Liquid biopsies	Glioblastoma
Exome studies—fusion detection^89^	Minimal	95-99%	Best Available	Multiple malignancies incl glioblastoma
Exome studies—molecular subtype classification^89^	Yes	50-80%	Fresh Frozen	
Single-cell RNA sequencing^90^	NA	NA	Fresh tissues or frozen cell suspension	Glioblastoma
Single-cell DNA sequencing^91^	NA	NA	Fresh tissues or frozen cell suspension	Glioblastoma
Spatial assays/spatial transcriptomics^92^	No	Greater preservation in FFPE	FFPE	Amyotrophic lateral sclerosis
Cell culture^93^	NA	NA	Fresh tissues	Diffuse intrinsic pontine glioma
Patient-derived xenografts (PDX)^94^	NA	NA	Fresh tissues	Pediatric midline glioma
Organoids^95^	NA	NA	Fresh tissues/ established cell lines	Glioblastoma

The data presented in Table 1 indicates that access to fresh and fresh frozen samples is essential for best practice research and for future research protocols. That said, in many cases, optimization can increase the sample fidelity of FFPE, particularly with respect to increased sequence depth for NGS assessment of DNA methylation^84^ or normalization to compensate for RNA degradation in RT-qPCR.^83^ Given the variability in sample requirements and the continual evolution of technology, tissue preservation approaches should be guided by research modalities and associated understandings of sample fidelity. Due to the limitations of surgical resection and the difficulties associated with collection of a dedicated research specimen, the opportunity to collect both FFPE and fresh frozen tissues in the postmortem setting is hugely advantageous.

**Figure 1.**
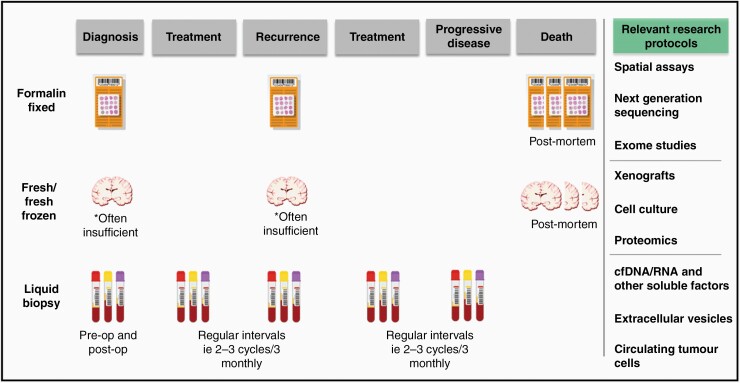
Suggested workflow for a comprehensive sample collection protocol and associated research methodologies. While fresh/fresh frozen tissue samples have been included at multiple ante-mortem time points, opportunities for collection of designated research specimens in this setting are limited due to the need to maximise sample for diagnostic purposes.

The development of a multi-factorial model of sample collection that will ensure future samples collected and stored are sufficient to allow the synergy of molecular and structural studies across various modalities is presented in Figure 1. While we maintain the view that postmortem tissue specimens are an essential resource for GBM research, an optimal workflow for sample collection and storage, such as that depicted in Figure 1, should include a range of sample types and methods to ensure a complete and high research utility resource.

Long-term storage and freezing protocols are an additional consideration with respect to sample fidelity for fresh frozen tissues. Snap freezing in isopentane, liquid nitrogen, or between precooled Teflon coated aluminum plates are approaches that have been extensively and successfully employed by those banking for neurodegenerative research such as the Columbia New York Brain Bank.^96,97^ Blocks obtained for storage in a fresh frozen capacity are placed between two precooled aluminum coated plates, –70 to –100°C, before being transferred to a tank containing liquid nitrogen vapor (LNV). LNV is favored over LN_2_ as it prevents fragmentation, reduces the leidenfrost effect, and enhances the cryogenic process by exerting less stress on the tissue. Tissues are then moved for long-term storage at –80°C.^97^

Ensuring sample size and storage conditions are appropriate for retrospective and unspecified research protocols is an enduring challenge, particularly given the degenerative impact of freeze/thaw cycles and the time taken to accumulate sufficient collections. Numerous studies have indicated that high-quality RNA can be extracted from tissue stored long-term at −80°C, however, signs of degradation can be observed in fresh frozen brain samples at 19–24 months, and RNA fragmentation can occur after 5 years at −70°C or −80°C.^98^ Once again, data is discordant and interpretation must be considered with respect to the variation in freezing protocols. Sample viability studies for the Columbia method described above could not be located, however, data from the NIH NeuroBiobank provides insight. This program uses isopentane for snap freezing allowing for better preservation of tissue morphology and greater research utility for analyses aligning structural and molecular profiles.^99^ Consequently, results may not be transferable between programs, yet promisingly, White et al. (2018) assessed frozen brain tissue stored for up to 23 years and identified no significant changes in RIN during storage time.^100^ Given the prevailing variability in protocols and lack of consistent long-term data paired with specific protocol, critical assertions of long-term viability are problematic and remain an enduring challenge in brain banking.

## How Does Postmortem Interval Impact Tissue Quality and are There Other Limitations in the Collection and Use of Postmortem Tissues?

Postmortem interval (PMI) and the extent to which PMI impacts tissue quality remains one of the largest potential limitations in the use of postmortem tissues given the impact on time-sensitive molecular changes. The PMI is calculated from the time of death through to final storage of tissue—typically freezing or fixation in formalin. The true impact of PMI on tissue quality is debatable and has led to an inconsistent definition of “rapid autopsy”. The majority of studies informing this conversation have been conducted in the neurodegenerative or psychiatric disease research space and therefore do not encompass the clinical hallmarks of death due to primary brain tumor, though notably no data directly comparing intraoperative and postmortem sample quality or utility in these settings could be identified. Further work on comparing paired intraoperative and postmortem samples, particularly when obtained from the same patient, would greatly enrich the field of understanding.

Consensus across United Kingdom biobanks suggests that a PMI of >72 hours is routine practice for use in genotyping, various arrays, and RT-PCR. This view is supported by publications documenting successful extraction of high-quality DNA, RNA, and protein from banked samples.^101,102^ While promising, this data is problematic for brain cancer research and inconsistent across the literature. Factors such as hypoxia, agonal state, systemic temperature, and systemic pH are frequently noted within the literature as factors of concern and potential limitations in the use postmortem tissues. Unfortunately, specific data quantifying and qualifying the presence and extent of the impact of each of these factors on postmortem brain cancer tissues is largely absent outside discussions of first principles.

Based on the literature,^101,102^ it can be accepted that high-quality molecular species are attainable, however, it is largely unknown to what extent the leeching of metabolites in a postmortem setting compromises the integrity of these samples as PMI values are extended. Furthermore, these data cannot necessarily be extrapolated to a brain cancer context given that PMI is exacerbated by disease-specific factors such as those summarized in Table 2.

**Table 2.** Comparison of Variables Impacting Tissue Quality and Serving as Potential Limitations to Research Use in Brain Cancer vs Neurodegenerative Diseases

Variable impacting tissue quality	Neurodegenerative diseases	Brain cancer
Intracranial pressure	Decreasing brain mass due to brain atrophy and neuronal death	Increasing brain tumor mass leading to rise in intracranial pressure^103^
Hypoxia	Characterized in hypoxic/vascular dementia^104^ and poststroke Alzheimer’s disease^105^	Increasing levels of hypoxia with both functional and pathological implications^106^
Prolonged agonal state and subsequent lowered pH^107^	Dyspnea reported in both Dementia and amyotrophic lateral sclerosis.^108^	Reduced consciousness, respiratory distress, pneumonia and agonal breathing (death rattle) common during prolonged terminal phase^108,110^
	Bronchopneumonia most common cause of death in Alzheimer’s disease^109^	
Necrosis	Necrotic and apoptotic pathways characterized in Alzheimer’s disease. Patchy foci of necrosis characterized^111^	Necrosis recognized as a hallmark feature of glioblastoma^113^
	Apoptosis primary mechanism of cell death in Parkinson’s disease.^112^	
Hyperpyrexia^114^	Largely absent unless indicative of infection	Malignant fever or paraneoplastic fever associated with both primary^115^ and secondary brain cancers^116^

As with preservation methods, the relevance of PMI data should be evaluated with respect to corresponding assays and research modalities—interpreted with caution given the lack of specific data from brain cancer models. Table 3 summarizes a selection of key components for investigation in postmortem tissues along with existing data relating to the impact of PMI on viability. Such data is discordant with the previously mentioned 72-hour model and may be the result of compounding metabolic or disease-specific factors. This is by no means an exhaustive list, but rather a representative sample to demonstrate the widespread viability of postmortem tissues collected through rapid autopsy.

**Table 3.** Impact of Postmortem Interval on Key Components Assessable in Postmortem Tissues

Molecule class/Methodology	Considerations relating to postmortem interval	Disease model
DNA	DNA quality may not be impacted by postmortem delay but rather by pH.^11,101^ DNA quality sufficient for whole genome/whole exome sequencing at 35hrs PMI^94^ High molecular weight DNA extracted from brain tissue 20 days postmortem^11^ (Data obtained from brain tumor and neurodegenerative models)	Brain tumor
		Non-specific Neurodegenerative
Gene expression	Aberrations (both increase and decrease) in expression with increased PMI^117^ largely due to hypoxia—need for normalization. Mean PMI 30hrs^118^	Control tissue/no known pathology
RNA/mRNA/miRNA	PMI of 3-4 hours optimal for miRNA and mRNA analysis.^119^ 82% samples RIN >7 at 3 hours, 42% RIN 7.5 at 7.7 hours.^117^ RIN >4 up to 36 hours (note RIN may not reflect the integrity of specific mRNAs)^100^	Control tissue/no known pathology
DNA methylation	No statistically significant differences in global methylation (5mC) were noted prior to a PMI of 9 hours. Consistent for hydroxymethylation (5hmC)^120^	Animal model (Rat)
Proteomics/phosphoproteomics^85–87^	Hypoxia induces phosphorylation changes but not global protein levels^85^ Consistent protein levels (ECE-2, KLK6, FVIII) noted between paired samples ranging from 0–72 hours in Alzheimer’s models^121,122^	Brain tumor
		Alzheimer’s disease
Cell culture^93^	Recommended 6–8 hours PMI (media), 24hrs to culture to maintain integrity of original tumor,^93^ however while best practice to minimize PMI to maintain fidelity of model, PMI does not appear to influence successful generation of cell lines or PDX models yet may introduce variations in expression.^94^	Brain tumor (pediatric)

Despite the promising data above, it remains conceivable that PMI influences tumor cell-intrinsic variations and the biological features of immune infiltrates within the tumor microenvironment.^123^

Interestingly, while PMI has long been considered the primary marker of quality and largest limitation in collecting postmortem tissues, this view is now challenged by a surge in publications suggesting that a lowered brain pH^11,101^ is a more accurate maker of quality. A definitive cause for variations in brain pH has not yet been elucidated and this remains an enduring limitation of postmortem collections, however hypoxic states, as discussed in Table 2 and the role of glycolysis may be implicated. This may be a key factor contributing to the discordance between PMI data. A more complete understanding of the necessary parameters for effective “rapid” autopsy in the brain cancer space is needed to ensure that protocols implemented mitigate factors such as hypoxia and cell stress. Overcoming these challenges and limitations can only be achieved with additional support for brain cancer postmortem programs.

The challenges associated with overcoming temporal heterogeneity are inversely true with respect to postmortem samples and serve as an additional limitation. While postmortem samples provide insight into the finite form of an individual’s tumor, additional specimens captured at multiple timepoints are needed to achieve a more complete picture of the temporal dynamics of tumor progression and treatment failure. Figure 1 provides a suggested collection pathway that incorporates the capture and preservation of serial blood samples. Blood specimens are typically processed and stored as plasma, serum, buffy coat, or peripheral blood mononuclear cells (PBMCs) to facilitate several important research questions. Firstly, PBMCs are a ready source of a patient’s “control” DNA that can provide a baseline of somatic gene alterations to allow the characterization of tumoral mutations. Secondly, archived longitudinal blood specimens are essential for the development of GBM liquid biopsies for the implementation of precision medicine and guiding patient-tailored therapies. Blood-derived analytes, such as circulating tumor cells (CTCs), free DNA (cfDNA), and RNA (cfRNA), EVs, and tumor-educated platelets (TEP) can be isolated and preserved and provide a “snapshot” of GBM as a systemic disease. GBM tumors release large quantities of EVs, which carry tumor-derived molecules across the blood-brain-barrier (BBB) into the circulation where they are stable and readily accessible. EV-associated biomarkers identified in patient blood has shown exciting promise for assessing the molecular state of GBM tumors in situ and may be used as a proxy for tumor tissue, allowing early diagnosis, providing objective measures of tumor activity and facilitating accurate tumor surveillance.^124–127^ Despite stability and BBB permeability issues, cfDNA may also prove to be an effective prognostic tool and surrogate marker of tumor burden.^128^ Indeed, preliminary data indicates that higher levels of preoperative cfDNA are positively correlated to reduced progression free survival,^128^ however there appears to be a diverse spectrum and mutational profile for GBM.^128,129^ When assessed against data obtained from postmortem samples, archived blood specimens may provide a complete and temporally dynamic opportunity to characterize brain tumors, from tumorigenesis to end-stage disease, overcoming temporal limitations and providing insight for translational research and improved patient outcomes.

## What are the Enduring Obstacles to Collecting Postmortem Brain Tissues and How Can we Overcome Them?

While the potential for postmortem brain tissues to further our understanding of brain tumor biology is considerable, acquisition of such samples is not without challenges. Postmortem donation programs, particularly those with a rapid autopsy framework, are resource-intensive, and pose numerous challenges related to logistics, infrastructure expenses, and expertise. In many cases, successful coordination of a donation can require after-hours health services, general practitioners, patient transport services, autopsy facilities, and biobanking staff—all with 24-hour availability.^130^ Available data within the existing body of literature pertaining to the overall cost of postmortem brain banking is both variable and somewhat outdated. Data from the Netherlands suggests the overall cost of brain banking is approximately €7,000–€15,000 per brain,^131^ however data from the United States suggests the figure is closer to $10,000–$30,000 USD and almost entirely related to personnel salaries of biobank staff, pathologists and mortuary attendants.^132^

Donor transport is an additional factor to consider, both in terms of resource expenditure and coordination—particularly in cases where donors die a considerable distance from the brain banking institution. While not unique to the Australian setting, this is particularly relevant for biobanking programs operating in countries such as Australia, given the vastness of the geography and consolidation of health services to metropolitan areas—particularly end-of-life services. As we have demonstrated through our own brain donation program, however, with extensive contingency planning and a healthy dose of altruism, geographical and logistical challenges associated with long-distance rapid autopsy programs can be overcome.^133^ Past successes aside, these challenges are not insignificant and as recognition of the value of postmortem specimens increases so too does the demand for such resources—further highlighting the need for greater investment and infrastructure support for postmortem brain donation programs. This also poses specific challenges with respect to modalities such as when working with subsequent cell lines and xenografts given the frequency of donation occurring outside traditional working hours. While planning can mitigate potential impacts of PMI, when considered in respect to logistics it can present as a limitation of postmortem tissues for some research modalities.

## Conclusion

In the past decade, there has been limited improvement in the treatment or outcomes of glioblastoma and further progress cannot be achieved without the highest quality biospecimens captured at clinically relevant time points to provide a complete picture of disease at primary presentation, recurrence, and death. Procurement and preservation of such samples is dependent on ongoing support for brain donation programs and greater advocacy and uptake of these samples from the research community for translational research. Resulting from a synergy between patients, researchers, and the medical community; postmortem brain banks present a flagship opportunity for research and may be a key to unlocking our understanding of brain tumor biology and ultimately improving outcomes for GBM patients.

## Notes

**Authorship Statement**. Research, analysis: C. G., J. L. Preparation of draft manuscript: C. G. Preparation of figures: C. G. Review and revisions to draft : C. P., K. A., H. H., M. W., J. L. Review and final approval: C. G., C. P., K. A., H. H., M. W., J. L. There are no unpublished papers included in this review.
